# Correlation between self-regulatory fatigue and physical activity in lung cancer patients undergoing comprehensive treatment

**DOI:** 10.1371/journal.pone.0341077

**Published:** 2026-01-16

**Authors:** Qiaoling Li, Jing Zhang, Shasha Meng, Fengxiang Tian, Qinqin Mei, Hui Wang, Hong Qi

**Affiliations:** 1 School of Clinical Medicine and The First Affiliated Hospital of Chengdu Medical College, Chengdu, Sichuan, China; 2 Department of Nephrology, The First Affiliated Hospital of Chengdu Medical College, Chengdu, Sichuan, China; 3 School of Nursing, Chengdu Medical College, Chengdu, Sichuan, China; 4 Department of Nursing, The First Affiliated Hospital of Chengdu Medical College, Chengdu, Sichuan, China; 5 Office of the Party Committee, the First Affiliated Hospital of Chengdu Medical College, Chengdu, Sichuan, China; Sichuan University WCSUH: Sichuan University West China Second University Hospital, CHINA

## Abstract

**Background:**

Self-regulated fatigue is often assessed in studies of chronic diseases. Research is needed on the self-regulation of fatigue and physical activity in lung cancer patients undergoing treatment, and the impact of these factors on this population.

**Objective:**

The goal of this study is to investigate the current status, influencing factors, and correlation between self-regulatory fatigue and physical activity in lung cancer patients undergoing comprehensive treatment.

**Methods:**

We used a convenience sampling method to enroll 188 lung cancer patients admitted to two tertiary hospitals in Chengdu from October 2024 to April 2025. Data were collected using a general information questionnaire and two scales: the Self-Regulatory Fatigue Scale (SRF-S) and The International Physical Activity Questionnaire-long form (IPAQ-L).

**Results:**

The mean self-regulatory fatigue score was 42.19 ± 9.06. The total metabolic equivalent (MET) of physical activity was 544.00 (0.00, 1386.00) MET-min/week, with leisure-time activity accounting for 429.00 (0.00, 1188.00) MET-min/week (data presented as median and interquartile range). Significant negative correlations were observed between Self-Regulatory Fatigue total scores and energy expenditure from housework, leisure activities, as well as total physical activity expenditure. Furthermore, self-regulatory fatigue was negatively correlated with both moderate-intensity and low-intensity physical activity, but positively correlated with high-intensity physical activity (*P* < 0.05). Multiple linear regression analysis identified gender, physical activity intensity, and pre-diagnosis exercise habits as significant independent influencing factors of self-regulatory fatigue in these patients (*P* < 0.05), collectively explaining 30.6% of the total variance (*R²* = 0.306).

**Conclusion:**

Engaging in appropriate leisure and household activities at moderate-to-low intensity may help alleviate the severity of self-regulatory fatigue in lung cancer patients undergoing comprehensive treatment. Healthcare providers should encourage appropriate activity to reduce the psychological burden and conserve self-regulatory resources.

## 1. Background

Primary bronchogenic carcinoma (hereafter referred to as lung cancer) represents the leading cause of cancer-related morbidity and mortality among malignant neoplasms in China [1]. Characterized by insidious onset with minimal early symptoms, this malignancy demonstrates aggressive biological behavior and rapid disease progression [2], with 57% of cases presenting with metastatic involvement at initial diagnosis [3].

Comprehensive treatment, which involves two or more therapeutic modalities such as combined radiotherapy and chemotherapy [4], can create synergistic effects and enhance treatment outcomes. While this paradigm enhances therapeutic efficacy through synergistic mechanisms, it concomitantly increases treatment-related toxicities [5].

Furthermore, patients undergoing multimodal therapy often require longer treatment durations due to disease complexity. The resulting pattern of frequent hospital admissions and discharges can diminish their psychological resilience, lead to a depletion of self-regulatory resources, and ultimately result in self-regulatory fatigue, adversely affecting their overall physical and psychological well-being [6,7].

Self-regulation fatigue refers to the depletion of psychological resources and the exhaustion of self-regulation capacity, commonly observed in patients experiencing prolonged stress, emotional distress, or decision fatigue [8].Self-regulatory fatigue, conceptualized as a persistent state of exhaustion resulting from chronic depletion of psychological control resources [8], serves as a validated metric for assessing psychological resource expenditure in chronic disease populations [9].

Cancer-related fatigue (CRF) refers to a distressing physiological and pathophysiological state resulting from the cancer itself or its treatment. It is fundamentally distinct from self-regulatory fatigue in both nature and underlying mechanisms [10]. Research indicates that engaging in low- to moderate-intensity physical activity can better promote physical and mental relaxation, thereby enhancing self-regulatory capacity and reducing fatigue [11]. Emerging evidence substantiates that structured physical activity interventions can ameliorate cancer-related symptoms, prolong survival duration, and mitigate negative affect states including cancer-related fatigue [12]. Physical activity encompasses any energy-consuming movement produced by skeletal muscles, performed across a spectrum of intensities—commonly classified into three levels—and accumulated through occupational, domestic, transportation, or leisure-time domains [13].

Numerous studies, both domestic and international, have investigated the factors influencing physical activity and their correlations in lung cancer patients following surgery or chemotherapy [12,14]. However, research remains scarce concerning the factors affecting both self-regulatory fatigue and physical activity, as well as their interrelationship, specifically among patients undergoing comprehensive treatment.

Employing a cross-sectional design, this study assessed self-regulatory fatigue and physical activity levels in lung cancer patients undergoing comprehensive treatment. It further examined the relationship between these variables to provide an empirical basis for developing targeted interventions aimed at enhancing self-regulatory capacity and increasing physical activity in this population.

## 2. Materials and methods

### 2.1 Participants and recruitment

Lung cancer patients admitted to the inpatient departments of two tertiary hospitals in Chengdu City from October 2024 to April 2025 were selected for the study by convenience sampling method. This study was reported in accordance with the STROBE guidelines and ensured that the content of the manuscript was consistent with the relevant project.

Both participating hospitals are tertiary Grade A medical institutions, serving as major healthcare centers in two separate administrative districts of Chengdu, which contributes to their regional representativeness. As regional referral centers, they attract a patient population that includes both urban and rural residents, enhancing the sociodemographic diversity of the sample. Moreover, these hospitals are equipped to provide multimodal comprehensive anticancer therapy, thereby meeting a key inclusion criterion of the study. Although the use of a non-random sampling method introduces certain limitations, the distribution of key clinical variables—such as disease stage and treatment modalities—within the sample was largely consistent with the national epidemiological characteristics of lung cancer.

The following individuals are eligible for inclusion: (1) Diagnosed with lung cancer in accordance with the Clinical Diagnosis and Treatment Guidelines for Lung Cancer of the Chinese Medical Association (2023 edition); (2) aged ≥18 years; (3) receiving comprehensive treatment with two or more therapies; and (4) possessing a certain level of communication ability. Exclusion standards: (1) Patients with impaired cognitive function or suffering from psychiatric disorders; (2) those with other malignant tumors or those who had previously suffered from cancer; and (3) those who had suffered from other traumatic events (such as natural or manmade disasters, divorce and widowhood, etc.) in the past month.

### 2.2 Ethical statement and patient consent

The study passed the ethical standards set by the Hospital Ethics Committee. It was approved by the committee (2024CYFYIRB-BA-Nov06). The patients gave informed consent to participate in this investigation voluntarily.

### 2.3 Measures

The questionnaire consisted of three parts: a general information survey, the Self-Regulatory Fatigue Scale(SRF-S), and the long form of the International Physical Activity Questionnaire (IPAQ-L).

#### 2.3.1 The general information survey.

This section included questions about the patient’s gender, age, occupation, marital status, educational level, residence, average monthly income, method of paying for medical insurance, body mass index (BMI), smoking status, disease diagnosis, disease stage, duration of illness, knowledge of exercise, sources of exercise information, exercise needs, and pre-illness exercise habits.

#### 2.3.2 Self-regulatory fatigue scale.

The Chinese version of the SRF-S was culturally adapted and validated by Wang Ligang et al. [15]. This 16-item instrument assesses three domains (cognitive, behavioral, and emotional regulation) using a 5-point Likert scale. Total scores range from 16 to 80, with higher scores indicating greater SRF severity. The maximum score for each domain is 30 (cognitive), 25 (emotional), and 25 (behavioral). The scale demonstrates good internal consistency (Cronbach’s α = 0.84).

#### 2.3.3 The international physical activity questionnaire-long form.

IPAQ-L was originally developed by the IPAQ Working Group and linguistically and culturally adapted into Chinese by Qu et al. in 2004 [13]. This validated instrument demonstrates robust psychometric properties, with test-retest reliability coefficients ranging from 0.67 to 0.93 and criterion validity between 0.62 and 0.82. Its use has been extensively documented in oncology research for evaluating physical activity patterns in cancer patients [16,17]. The 25-item questionnaire systematically evaluates four domains of physical activity during the preceding seven days: occupational activities, transportation-related activities, household chores, and leisure-time exercise. Each domain captures activity intensity through three distinct levels: Low, moderate, and vigorous. Energy expenditure is derived by applying the metabolic equivalent (MET) value corresponding to the intensity of each activity. The weekly volume of activity-specific expenditure, expressed in MET-minutes, is calculated as follows: MET-min/week = MET value × duration (minutes per day) × frequency (days per week). The total physical activity expenditure is then obtained by summing the MET-min/week values across all activity domains.

### 2.4 Sample size calculation

The sample size was calculated using Kendall’s estimation method [18], which recommends a sample size of 5–10 times the number of research variables. This study included 21 variables (17 items from the general information questionnaire, 3 dimensions from the Self-Regulatory Fatigue Scale, and 1 dimension from IPAQ-L). Accounting for a potential 20% attrition rate, the minimum required sample size was calculated as Nmin = 21 × 5×(1 + 20%) = 126, and the maximum sample size as Nmax = 21 × 10×(1 + 20%) = 252.

### 2.5 Study procedure

Prior to data collection, the research team conducted a comprehensive orientation session for participants, during which they delineated the study’s objectives, procedures, and confidentiality safeguards. Following the acquisition of written informed consent through the completion of signed consent forms, participants proceeded to complete the survey via a secure online platform. To ensure the integrity of the collected data, the investigators provided real-time clarification of the questionnaire items upon the request of the participants. This clarification was provided throughout the administration process. The average completion time was standardized at 10 minutes per questionnaire to maintain procedural consistency. Subsequent to the submission process, the research staff undertook quality control checks to ascertain the thoroughness of the responses prior to the analysis of the data.

Initially, 200 questionnaires were distributed; however, only 188 met the inclusion criteria for analysis, resulting in a 94.0% valid response rate. The high rate of participation observed in this study is indicative of two factors. Firstly, the survey administration method employed was characterized by a high degree of methodological rigor. Secondly, the level of engagement exhibited by the participants was notably robust.

### 2.6 Data analysis

Statistical analyses were conducted using SPSS 26.0, with a significance level set at *α* = 0.05. The normality and homogeneity of variance were assessed using histograms and *Q-Q* plots to ensure the appropriateness of parametric tests. Descriptive statistics were used to summarize the sociodemographic characteristics, disease-related data, and other variables, expressed as frequencies and percentages.SRF-S scores were presented as mean ± standard deviation (*SD*) or median and interquartile range (*M [P25, P75]*), depending on the distribution of the data.Univariate analysis of SRF in lung cancer patients was conducted using independent *t*-tests or one-way *ANOVA*, as appropriate. *Spearman’s* correlation test was utilized to investigate the association between SRF and physical activity levels. A multiple linear regression analysis was conducted to examine the determinants of self-regulatory fatigue among lung cancer patients receiving comprehensive treatment. A p-value less than 0.05 was considered statistically significant.

## 3 Results

### 3.1 Analysis of SRF in lung cancer patients undergoing comprehensive treatment

The mean SRF-S among lung cancer patients was 42.19 ± 9.06. The subscale scores, ranked from highest to lowest, were as follows:Cognitive dimension: 18.28 ± 3.67; Emotional dimension: 12.94 ± 3.85; Behavioral dimension: 10.97 ± 3.14.(Refer to Table 1 for detailed data.)

**Table 1:** Status of Self-Regulated Fatigue in Lung Cancer Patients Undergoing Comprehensive Treatment (n = 188).

Dimension	Mean Score ($\overset{―}{X}$±S)	Score/Time Frame
Total Self-Regulated Fatigue Score	42.19 ± 9.06	16 ~80
Cognitive Dimension	18.28 ± 3.67	6 ~ 30
Emotional Dimension	12.94 ± 3.85	5 ~ 25
Behavioral Dimension	10.97 ± 3.14	5 ~ 25

### 3.2 Univariate analysis of SRF in lung cancer patients undergoing comprehensive

The present study encompassed a total of 188 lung cancer patients receiving comprehensive treatment, with 116 males (61.7%) and 72 females (38.3%) constituting the sample. Female patients demonstrated significantly higher levels of SRF compared to male patients. Furthermore, the majority of patients (67.9%) were aged >60 years, indicating that the majority of diagnosed cases were elderly. Univariate analysis revealed statistically significant differences *(P* < 0.05) in SRF’s based on the following: gender, smoking status, disease diagnosis, disease stage, pre-illness exercise habits, knowledge of exercise, and perceived necessity of exercise education.(Refer to Table 2 for detailed statistical comparisons.)

**Table 2:** Univariate Analysis of Self-Regulated Fatigue in Lung Cancer Patients Undergoing Comprehensive Treatment (*n*  =  188).

	Items	*n(%)*	Self-Regulated Fatigue ($\overset{―}{X}$*±S*)	*t/F*	*P*
Gender	Male	116 (61.70)	40.81 ± 8.34	−2.687^*^	0.008
	Female	72 (38.30)	44.40 ± 9.76		
Age	[18, 50]	10 (5.30)	45.20 ± 7.02	0.582	0.560
	(50, 60]	47 (25.00)	42.04 ± 9.43		
	>60	131 (67.90)	42.01 ± 9.08		
Education Level	Primary school or below	95 (50.50)	42.23 ± 9.28	1.079	0.368
	Junior high school	61 (32.40)	41.13 ± 9.25		
	Senior high school	21 (11.20)	42.38 ± 7.20		
	Associate degree	7 (3.70)	47.29 ± 6.55		
	Bachelor’s degree or above	4 (2.10)	47.25 ± 12.37		
Occupation	Employed	13 (6.90)	42.23 ± 10.95	0.114	0.952
	Retired	111 (59.00)	42.49 ± 8.99		
	Unemployed	53 (28.20)	41.70 ± 9.01		
	Farming	11 (5.90)	41.45 ± 8.71		
Marital Status	In a relationship	173 (92.00)	41.95 ± 8.95	−1.196*	0.233
	Single (included divorced or widowed)	15 (8.00)	44.87 ± 10.17		
BMI	<18.5	24 (12.80)	45.29 ± 9.44	2.078	0.105
	[18.5, 24.0)	103 (54.80)	42.24 ± 8.79		
	[24.0 ~ 28.0)	50 (26.60)	41.64 ± 8.75		
	>28.0	11 (5.90)	37.36 ± 10.72		
Residence Type	Urban	158 (84.00)	41.78 ± 9.27	−1.398^*^	0.164
	Rural	30 (16.00)	44.30 ± 7.61		
Medical Insurance Type	Urban Employee Basic Medical Insurance	151 (80.30)	41.91 ± 9.26	0.381	0.683
	Urban-Rural Resident Basic Medical Insurance	34 (18.10)	43.18 ± 8.36		
	Out-of-pocket	3 (1.60)	44.67 ± 7.51		
Family income per month	<¥2,000	45 (23.90)	42.87 ± 8.33	1.170	0.313
	¥2,000-¥5,000	112 (59.60)	42.54 ± 9.11		
	¥5,001-¥10,000	31 (16.50)	39.94 ± 9.82		
Smoking Status	Never smoker	119 (63.30)	43.31 ± 9.33	4.197	0.016
	Former smoker	50 (26.60)	39.06 ± 7.69		
	Current smoker	19 (10.10)	43.37 ± 9.13		
Diagnosis	Small cell lung cancer	23 (12.20)	44.91 ± 7.98	3.551	0.031
	Adenocarcinoma	112 (59.60)	42.84 ± 9.37		
	Squamous cell carcinoma	53 (28.20)	39.62 ± 8.36		
Disease Stage	Stage I	15 (8.00)	42.20 ± 8.03	3.502	0.017
	StageⅡ	14 (7.40)	38.50 ± 7.38		
	StageⅢ	37 (19.70)	38.92 ± 8.16		
	StageⅣ	122 (64.90)	43.60 ± 9.32		
Disease Duration	<1 year	92 (48.90)	41.20 ± 8.18	1.083	0.341
	1-5 years	82 (43.60)	43.10 ± 10.01		
	>5 years	14 (7.40)	43.36 ± 8.56		
Pre-Diagnosis Exercise Habits	Never exercise	8 (4.30)	45.25 ± 7.36	9.533	<0.001
	1-2 times per week	3 (1.60)	54.00 ± 9.54		
	3-4 times per week	13 (6.90)	51.54 ± 10.81		
	5-6 times per week	53 (28.20)	44.30 ± 8.40		
	Daily exercise	111 (59.00)	39.54 ± 8.01		
Level of Knowledge About Exercise	No knowledge	21 (11.20)	47.76 ± 8.38	7.602	0.001
	Some knowledge	74 (39.40)	43.28 ± 9.00		
	Moderate knowledge	93 (49.50)	40.05 ± 8.63		
Perceived Need to Learn About Exercise	Neutral	31 (16.50)	45.48 ± 9.58	3.670	0.027
	Necessary	146 (77.70)	41.24 ± 8.96		
	Unnecessary	11 (5.90)	45.45 ± 5.92		
Sources of Exercise Information (Media)	None	139 (73.90)	41.63 ± 9.18	−1.433^*^	0.154
	Media	49 (26.10)	43.78 ± 8.60		
Sources of Exercise Information (Healthcare Providers)	None	133 (70.70)	42.92 ± 8.99	1.730^*^	0.085
	Healthcare Providers	55 (29.30)	40.42 ± 9.06		
Sources of Exercise Information (Community Health Centers)	None	134 (71.30)	42.28 ± 9.07	0.232^*^	0.817
	Community Health Centers	54 (28.70)	41.94 ± 9.11		
Sources of Exercise Information (All of the Above)	None	80 (42.60)	41.79 ± 9.12	−0.518^*^	0.605
	All of the Above	108 (57.40)	42.48 ± 9.04		

Note: In statistical values, * indicates *t*-values, while all others are *F*-values.

### 3.3 Analysis of physical activity levels in lung cancer patients undergoing comprehensive treatment

Of the 188 patients, 4 (2.1%), 5 (2.7%), 28 (14.9%), and 115 (61.2%) reported engaging in occupational, transportation, household, and leisure-related physical activities, respectively. In terms of intensity, 5 (2.7%), 34 (18.1%), and 116 (61.7%) patients reported engaging in high-, moderate-, and low-intensity physical activities. These findings indicate that patients primarily engaged in leisure-related physical activities, with the overall activity level being generally low to moderate, and dominated by low-intensity activities. As the physical activity data did not conform to a normal distribution, the results were expressed as median and interquartile range (IQR). The subsequent analysis yielded the following findings: Total physical activity energy expenditure: The weekly MET-min/week value ranged from 0.00 to 1386.00. The subject of this investigation is the energy expenditure associated with leisure-time physical activity. The mean weekly MET-min/week expenditure was 429.00 (0.00, 1188.00), accounting for 67.4% of the total physical activity expenditure. The metabolic equivalents (METs) for physical activities of different intensities and types are presented in Table 3 and Table 4.

**Table 3:** Metabolic Equivalent (MET) Values by Intensity and Type of Physical Activity in Lung Cancer Patients Receiving Comprehensive Treatment (n  =  188).

Items	M (P25, P75)	Percentage of Total MET-minutes (%)
Physical Activity Domain		
Occupational Activity	0.00 (0.00, 0.00)	14.2
Transportation Activity	0.00 (0.00, 0.00)	1.6
Domestic Activity	0.00 (0.00, 0.00)	16.7
Leisure-Time Activity	429.00 (0.00, 1188.00)	67.4
Activity Intensity Level		
Vigorous Intensity	0.00 (0.00, 0.00)	13.1
Moderate Intensity	0.00 (0.00, 0.00)	25.1
Light Intensity	396.00 (0.00, 1188.00)	61.8
Total MET-minutes/week	544.00 (0.00, 1386.00)	100

**Table 4:** Physical Activity Levels in Lung Cancer Patients Undergoing Comprehensive Treatment (n = 188).

**Variable**	**Category**	**n**	**%**
Physical Activity Domain	Occupational Activity	4	2.1%
	Transportation Activity	5	2.7%
	Domestic Activity	28	14.9%
	Leisure-Time Activity	115	61.2%
	Total MET-minutes/week	123	65.4%
Activity Intensity Level	Vigorous Intensity	5	2.7%
	Moderate Intensity	34	18.1%
	Light Intensity	116	61.7%

### 3.4 Correlation analysis between SRF and physical activity in lung cancer patients undergoing comprehensive treatment

Spearman’s correlation analysis revealed significant negative correlations between SRF and specific domains of physical activity: The following categories of energy expenditure are of particular interest: household activity (*r* = −2.74, *P* < 0.05), leisure activity( (*r* = −5.08, *P* < 0.05)), and total physical activity (*r* = −5.14, *P* < 0.05). These negative correlations were observed for both the total SRF-S and its subscale scores (cognitive, emotional, and behavioral dimensions), indicating that increased SRF was associated with decreased physical activity levels in these domains. However, no statistically significant correlations were identified between SRF and the relationship between work-related activity energy expenditure and transportation-related activity energy expenditure. Regarding exercise intensity, moderate-intensity (*r* = −0.279, *p* < 0.05) and low-intensity (*r* = −0.522, *p* < 0.05) physical activities demonstrated significant negative correlations with self-regulatory fatigue total scores, whereas vigorous-intensity activity showed a significant positive correlation with self-regulatory fatigue (*r* = 0.208, *p* < 0.05). For a more comprehensive overview of the correlation coefficients and p-values, please refer to Table 5.

**Table 5:** Correlation Analysis Between Self-Regulated Fatigue and Physical Activity in Lung Cancer Patients Undergoing Multimodal Therapy (n = 188).

Items	Total Self-Regulated Fatigue Score	Cognitive Dimension	Emotional Dimension	Behavioral Dimension
Occupational Activity	0.028	−0.078	0.03	0.091
Transportation Activity	0.059	−0.107	0.08	0.126
Domestic Activity	−.274**	−.244**	−.245**	−.218**
Leisure-Time Activity	−.508**	−.593**	−.372**	−.345**
Vigorous Intensity	0.111	−0.045	0.116	.208**
Moderate Intensity	−.279**	−.254**	−.257**	−.209**
Light Intensity	−.522**	−.622**	−.381**	−.361**
Total MET-minutes/ week	−.514**	−.621**	−.396**	−.318**

Note. **P < 0.01, *P < 0.05.

### 3.5 Multivariate analysis of self-regulatory fatigue in lung cancer patients undergoing comprehensive treatment

Multiple linear regression analysis was performed with the total score of self-regulatory fatigue as the dependent variable and variables showing statistically significant differences in univariate analysis and correlation analysis as independent variables. The results indicated that gender, physical activity intensity, and pre-diagnosis exercise were significant independent factors influencing self-regulatory fatigue in lung cancer patients (*P* < 0.05), collectively accounting for 30.6% of the total variance (*R²* = 0.306). See Table 6.

**Table 6:** Multiple Linear Regression Analysis of Factors Influencing Self-Regulated Fatigue in Lung Cancer Patients Undergoing Multimodal Therapy (n = 188).

Factors	Regression Coefficient	Standard Error	Standardized Regression Coefficient (β)	*t*	*p*-value
(Constant)	43.696	1.931		22.623	<0.000
Physical Activity Level					
Moderate-intensity	−0.004	0.001	−0.363	−5.632	<0.000
Low-intensity	−3.510	1.195	−0.191	−2.938	0.004
Exercise before illness					
Exercised daily	−0.002	0.001	−0.199	−3.107	0.002
Gender	2.850	1.168	0.153	2.440	0.016

Note: F = 21.573, P < 0.001, R² = 0.320, Adjusted R² = 0.306.

## 4 Discussion

### 4.1 SRF in lung cancer patients undergoing comprehensive treatment

In this study, the self-regulatory fatigue score of lung cancer patients was 42.19 ± 9.06, which is higher than that reported in healthy populations (36.5 ± 8.9) [15] and peritoneal dialysis patients (41.78 ± 9.76) [19]. We speculate that this elevated level may be associated with prevalent misconceptions such as “cancerphobia” and “cancer equals death” among patients. Although this study did not measure relevant psychophysiological indicators, we hypothesize that heightened negative illness perceptions may correlate with increased fear of cancer recurrence. The experience of cancer disrupts their conception of health, which, in conjunction with the dearth of knowledge, misunderstanding, or over-interpretation of cancer, often precipitates fear of disease and self-regulation disorders, resulting in the impairment of patients’ self-regulation resources [20]. Nevertheless, these explanations remain indirect. Future studies should incorporate these psychological and physiological biomarkers to clarify their potential mediating roles. However, in contrast to the findings reported by Qi Cai lian [21], it is noteworthy that the majority of the patients included in this study were early-stage chemotherapy patients who experienced more pronounced adverse effects. In contrast, the patients in this study received a minimum of two treatments, and as they underwent repeated treatments, they demonstrated a gradual adaptation at both the physiological and psychological levels. Consequently, their acceptance levels were higher compared to those observed in earlier treatments, particularly in terms of the adverse effects that emerged in the earlier treatments.

The cognitive dimension scores (18.28 ± 3.67) in the study were higher than the behavioral (10.97 ± 3.14) and emotional (12.94 ± 3.85) dimension scores, which is consistent with the findings of previous studies [21,22]. The rationale behind this phenomenon is that the majority of lung cancer patients are elderly, and elderly lung cancer patients exhibit reduced tolerance and prolonged duration of adverse effects. Consequently, the risk of cognitive impairment is elevated, which can adversely impact the patient’s comprehension of the disease and their capacity for independent decision-making. In the results of Xu Min’s study [23], the behavioral dimension subscale score was the highest. This may be due to the fact that pregnant women with gestational diabetes mellitus (GDM) need to continuously invest physical and mental resources during the self-management of the disease. Long-term interventions such as dietary management, glycemic monitoring, and moderate physical activity require continuous, high-intensity self-restraint, which is prone to trigger behavioral control fatigue. Cross-condition comparisons are limited by the distinct clinical characteristics of lung cancer populations—such as unique treatment sequelae and prognostic uncertainty. Thus, future research on self-regulatory fatigue should be expanded specifically in this patient group.

### 4.2 Physical activity status of lung cancer patients undergoing comprehensive treatment

The median total physical activity level among lung cancer patients in this study was 544 MET-min/week, lower than the 602.5 MET-min/week reported by Li Lin [24]. Most participants in this study were retired lung cancer patients, so the discrepancy may be due to reduced organ function and resistance associated with advanced age. As a respiratory disease, lung cancer itself further restricts patients’ physical activity levels [25]. In this study, 59% of the participants were retired lung cancer patients. This demographic characteristic aligns with the typical age distribution of lung cancer, which is predominantly diagnosed in individuals aged 55–79 years. After retirement, these patients have less energy-consuming physical activity and work-related transportation. This leaves more leisure time, which means they are more likely to take part in activities they enjoy. As a result, they do the most physical activity in their free time [24].

The inclination towards leisure-time physical activity (LTPA) among the majority of patients corresponds with the observations reported by Bai et al. [26], who documented that augmented physical activity effectively enhances muscle strength, improves pulmonary function, mitigates cancer- and treatment-related adverse effects, and reduces disease burden.

A significant proportion of the lung cancer patient population, 61.7%, engaged in light-intensity physical activity (LIPA), a finding that aligns with prior research [27]. This activity pattern likely stems from cancer- and treatment-induced symptoms, such as fatigue and dyspnea, which diminish both motivation and capacity for higher-intensity exercise. Due to its accessibility, walking was identified as the preferred LIPA modality among the study participants.

Furthermore, age-related physiological decline contributed to progressive reductions in moderate-to-vigorous physical activity (MVPA) [28]. This phenomenon is indicative of the combined impact of natural aging processes and cancer-related functional deterioration on patients’ exercise tolerance.

### 4.3 Factors influencing SRF in lung cancer patients undergoing comprehensive treatment

#### 4.3.1 Gender differences.

Female patients demonstrated significantly higher levels of self-regulated fatigue in comparison to male patients, which is consistent with the findings reported by Wang et al.[8]. This discrepancy may be attributed to gendered societal roles, wherein women frequently bear the dual burden of domestic responsibilities and occupational demands, thereby exacerbating psychological stress. Furthermore, it has been observed that women often demonstrate greater emotional awareness and sensitivity to health-related changes. This heightened emotional reactivity may result in more pronounced emotional fluctuations when facing disease progression, potentially leading to increased self-regulatory fatigue [29]. Cyclical hormonal fluctuations intrinsic to female physiology may expedite the exhaustion of self-regulatory resources. In support of this perspective, Wang Yao et al. [30] demonstrated that hormonal fluctuations in females during specific physiological periods can predispose them to autonomic nervous system dysfunction. Subsequent negative emotional states may increase the burden on psychological regulation mechanisms, thereby exacerbating self-regulatory fatigue.

#### 4.3.2 Physical activity.

Research indicates an inverse association between physical activity intensity and the severity of self-regulatory fatigue. This relationship may be explained by the superior efficacy of moderate-intensity activity in minimizing declines in cardiopulmonary function and muscular strength, while most effectively alleviating both physical and mental fatigue. Although low-intensity exercise regimens still demonstrate significant benefits, their therapeutic scope and effect magnitude appear comparatively limited [31].

#### 4.3.3 Exercise.

Patients who had not engaged in physical activity prior to the onset of the disease, due to the physical exertion associated with the cancer itself, exhibited a greater utilization of their resources and a more significant decline in their ability to regulate themselves. Conversely, patients who had engaged in physical activity prior to the onset of their illness and were cognizant of the significance of exercise opted to partake in moderate physical exertion with the objective of ameliorating their physical and mental fatigue, alleviating their symptoms, and enhancing their quality of life subsequent to a cancer diagnosis [32].

### 4.4 Correlation analysis between SRF and physical activity in lung cancer patients undergoing comprehensive treatment

*Spearman’s* correlation analysis revealed significant negative correlations between self-regulatory fatigue and energy expenditure from housework *(r* = −0.274, *p* < 0.01), leisure activities (*r* = −0.512, *p* < 0.01) and total physical activity (*r* = −0.518, *p* < 0.01). One possible explanation is that patients experiencing more severe self-regulatory fatigue may be more susceptible to negative emotions and passive coping strategies, which reduces their engagement in physical activities. Conversely, those with milder fatigue may be better able to motivate themselves to participate in physical activities, which could help alleviate their physical burden.

Previous studies have indicated that patients undergoing treatment experience the dual burden of disease and treatment-related challenges. Higher levels of self-regulatory fatigue may lead to increasingly passive coping strategies during prolonged illness, resulting in reduced physical activity [33]. Supporting this, Sprod et al.[34] demonstrated that physical activity can alleviate physical and mental fatigue in cancer patients, enhancing self-regulatory capacity and reducing psychological resource depletion. Building on these findings, Sundgot-Borgen et al.[35] observed in their study of post-bariatric surgery patients that reduced physical activity can undermine self-regulatory capacity, potentially leading to psychological distress, maladaptive behaviours, and mental fatigue. Taken together, these studies suggest a complex interplay between self-regulatory fatigue and physical activity that may form a bidirectional relationship warranting further investigation in cancer populations.

A significant positive correlation was observed between vigorous-intensity activity and self-regulatory fatigue (*r* = 0.208, *p* < 0.01), while both moderate-intensity and low-intensity activities demonstrated significant negative correlations with self-regulatory fatigue total scores (*r* = −0.279 and *r* = −0.527, respectively, *p* < 0.01). This pattern may be explained by the potential of sustained vigorous exercise to induce physical and mental exhaustion, thereby compromising recovery capacity and potentially exacerbating self-regulatory fatigue [36]. In contrast, low-to-moderate intensity exercise may generate higher energy levels, promote psychological detachment and relaxation, and consequently enhance self-regulatory capacity while reducing fatigue [37]. Therefore, healthcare professionals should educate cancer patients on engaging in leisure activities, household chores and low-to-moderate intensity physical activities that are suitable for them. Such interventions have the potential to enhance self-regulatory capacity, alleviate psychological distress, promote treatment adherence, and encourage the implementation of standardized anticancer therapy, thereby contributing to an improvement in quality of life. As this study employed a cross-sectional design, it was unable to establish causality between self-regulatory fatigue and physical activity. Further longitudinal or interventional studies are needed to investigate the potential causal relationships and bidirectional effects between these variables.

## 5 Limitations

The present study is not without its limitations. The participants in this study were recruited from two hospitals in a single city, which may limit the representativeness of the sample to the broader population of lung cancer patients in China. Future research should expand the sampling framework to include diverse geographic regions (e.g., multiple cities or provinces) to enhance the generalizability of findings. Additionally, the sample predominantly consisted of older, retired patients, with limited representation of younger and working individuals. To address this, future studies could employ stratified sampling methods to ensure balanced inclusion across age groups and occupational statuses, thereby enabling a more nuanced exploration of the relationship between self-regulatory fatigue and physical activity in lung cancer patients undergoing comprehensive treatment. Conclusions drawn from this study should be interpreted with caution and are primarily applicable to older, non-working populations. Currently, research directly investigating self-regulatory fatigue in lung cancer patients undergoing comprehensive treatment remains limited. Although our findings share certain similarities with observations in other patient populations such as breast cancer patients or hemodialysis patients, the unique challenges faced by lung cancer patients—including disease-specific symptoms, treatment trajectories, and psychosocial burdens—suggest that the underlying mechanisms may differ significantly. Future studies should prioritize in-depth exploration of the psychological mechanisms specific to this population, particularly those receiving multimodal anticancer therapies.

While extant literature has extensively investigated SRF in chronic disease patients and healthy populations, research focusing on cancer populations remains underdeveloped. It is recommended that future investigations encompass a broader spectrum of cancer types to further enrich this field of study. Presently, research on SRF in patients receiving comprehensive cancer treatment is still in its preliminary stages. Subsequent studies should expand the scope to examine other cancer populations, thereby broadening our understanding.

## 6 Clinical implications

Our study examined self-regulation fatigue and its impact on lung cancer patients undergoing comprehensive treatment, as well as the correlation between self-regulation fatigue and physical activity. Self-regulation fatigue was heavier in women than in men, highlighting the need to alleviate self-regulation fatigue and increase clinical attention to women cancer patients. The greater the level of self-regulation fatigue in lung cancer patients on comprehensive treatment, the less likely they were to participate in physical activity. Therefore, we need to focus on patients’ psychological depletion resources to reduce physical and psychological burdens while increasing physical activity. This highlights the fact that reducing the depletion of self-regulatory resources is necessary to promote patients’ physical and mental health. The results of this study provide empirical evidence for the development of individualized intervention programs to reduce patients’ self-regulation fatigue and enhance their quality of life. Meanwhile, the findings not only provide theoretical guidance for clinical nursing practice, but also point out the direction for subsequent related research.

## 7 Conclusion

In summary, patients with lung cancer who undergo comprehensive treatment experience a form of SRF. The following factors have been identified as key influencers: gender, smoking status, disease diagnosis, disease stage, pre-diagnosis exercise habits, knowledge about exercise, and perceived need for exercise education. The predominant physical activity patterns among these patients were leisure-time and low-intensity activities.It provided a basis for future targeted intervention strategies.

## Supporting information

S1 File Supporting information. (ZIP)
