# Correction: Correlation between self-regulatory fatigue and physical activity in lung cancer patients undergoing comprehensive treatment

**DOI:** 10.1371/journal.pone.0346781

**Published:** 2026-04-07

**Authors:** Qiaoling Li, Jing Zhang, Shasha Meng, Fengxiang Tian, Qinqin Mei, Hui Wang, Hong Qi

The affiliation for the first, second, and seventh authors is incorrect. The affiliation for these authors is: School of Clinical Medicine and The First Affiliated Hospital of Chengdu Medical College, Chengdu, Sichuan, China.
